# Plant-pathogen interactions: making the case for multi-omics analysis of complex pathosystems

**DOI:** 10.1007/s44154-025-00260-7

**Published:** 2025-10-28

**Authors:** Sadegh Balotf, Richard Wilson, Roghayeh Hemmati, Mahsa Eshaghi, Calum Wilson, Luis A. J. Mur

**Affiliations:** 1 Centre for Crop Health, University of Southern Queensland, Toowoomba, QLD Australia; 2 Central Science Laboratory, University of Tasmania, Hobart, TAS Australia; 3 Department of Plant Protection, Faculty of Agriculture, University of Zanjan, Zanjan, Iran; 4 Department of Plant Biotechnology, Faculty of Agriculture, Tarbiat Modares University, Tehran, Iran; 5 Tasmanian Institute of Agriculture, University of Tasmania, Hobart, TAS Australia; 6 Department of Life Sciences, Aberystwyth University, Aberystwyth, UK

**Keywords:** Plant-pathogen interactions, Genomics, Transcriptomics, Proteomics, Metabolomics, Multi-omics

## Abstract

Understanding plant-pathogen interactions requires a systems-level perspective that single-omics approaches, such as genomics, transcriptomics, proteomics, or metabolomics alone, often fail to provide. While these methods are informative, they are limited in their ability to capture the complexity of the dynamic molecular interactions between host and pathogen. Multi-omics strategies offer a powerful solution by integrating complementary data types, enabling a more comprehensive view of the molecular networks and pathways involved in disease progression and defence. Although technological advances have made omics analyses more accessible and affordable, their integration remains underutilised in plant science. This review highlights the limitations of single-omics studies in dissecting plant-pathogen interactions and emphasises the value of multi-omics approaches. We discuss available computational tools for data integration and visualisation, outline current challenges, including data heterogeneity, normalisation issues, and computational demands, and explore future directions such as the exploitation of artificial intelligence-based approaches and single-cell omics. We conclude that the increasing accessibility and affordability of omics analysis means that multi-omics strategies are now indispensable tools to investigate complex biological processes such as plant-pathogen interactions.

## Introduction

Plants interact with a variety of different microorganisms. Microbial populations have different effects on the physiological activities of plants. Some interactions are beneficial while others are pathogenic (Schirawski and Perlin 2018; Crandall et al. 2020). In response to stress, cells trigger molecular cascades that include changes in gene expression, activation of signalling pathways such as the mitogen-activated protein kinase (MAPK) pathway, modulation of protein synthesis, induction of heat shock proteins (HSPs), and alterations in metabolite levels to support cellular adaptation and maintain homeostasis (Nicaise et al. 2009; Zhang et al. 2021). Central to understanding plant-pathogen interactions is the concept of a “pathosystem” which acknowledges that features of associated host plant and pathogen shift so they are distinct from when they are considered in isolation (Spanu et al. 2024).

**Fig. 1:**
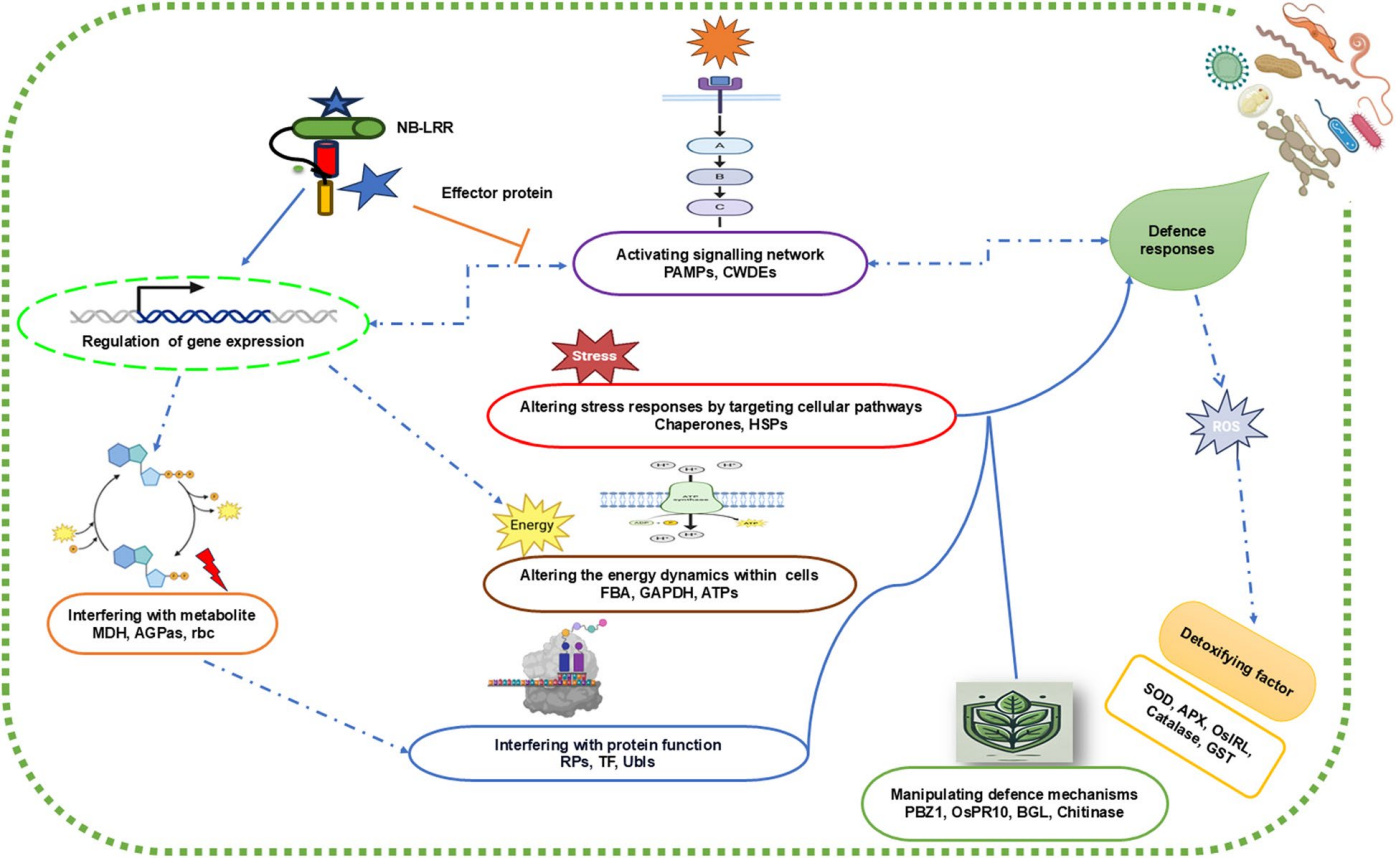
The complexity of plant-pathogen interactions signalling mechanisms and defence responses. Pathogen-associated molecular patterns (PAMPs) and cell wall-degrading enzymes (CWDEs) trigger a cascade of defence responses, involving multiple processes and key components. Gene expression is regulated through the activation of genes influencing metabolic processes, such as malate dehydrogenase (MDH), glucose 1-phosphate adenyltransferase (AGPas), and Rubisco (rbs). Effector proteins interact with plant defence components, including NB-LRR receptors (Nucleotide-Binding Leucine-Rich Repeat proteins)(“resistance genes”), triggering or suppressing specific defence responses. Stress responses are altered by targeting cellular pathways, such as the production of chaperonin proteins, heat shock proteins (HSPs), and other stress-related proteins to mitigate damage. Energy dynamics within cells are influenced by the upregulation of enzymes like fructose-bisphosphate aldolase (FBA), oxidoreductase, GAPDH, and ATP synthase, supporting cellular activities. Protein function is interfered with through the induction of ribosomal proteins (RPs), translational factors (TFs), and ubiquitin-like proteins (Ubls) to maintain cellular functions. Plant defence mechanisms are manipulated by upregulating specific defence-related proteins, including PR proteins like PBZ1, β−1,3-glucanase (BGL), and chitinase, to target pathogens. Detoxifying enzymes, such as superoxide dismutase (SOD), ascorbate peroxidase (APX), catalase, and glutathione S-transferase (GST), neutralise reactive oxygen species (ROS) to protect plant cells

The advent of the omics era commenced with the development of whole genome sequencing techniques. This led to research strategies that mainly focused on the study of entire genomes. However, unlike the relatively static genome, other omics layers (e.g., transcriptome, proteome, metabolome) are dynamic, and varying according to cellular/tissue localisation and the developmental stage of an organism (Griffin 2004). These omic layers therefore better reflect the changes occurring when two interacting partners come together to form the pathosystem, However, despite significant advancements in individual omics fields, each approach offers only a partial view of the complex biological systems involved in plant-pathogen interactions. Multi-omics approaches are particularly well-suited to studying pathosystems, as they enable the simultaneous profiling of both the host and pathogen (Allwood et al. 2010; Li et al. 2019a, b), revealing co-evolutionary patterns and regulatory networks that are often missed by single-omics approaches. Figure 1 illustrates the complexity of plant-pathogen interactions and highlights the molecular events that occur when a pathogen invades a plant host.

This review aims to highlight the essential role of integrative multi-omics approaches in advancing our understanding of plant-pathogen interactions. By integrating transcriptomics, proteomics, and metabolomics, researchers can identify key regulatory molecules, decipher effector functions, and uncover mechanisms of disease resistance or susceptibility. The review also focuses on the computational tools that facilitate the integration of these diverse datasets, ranging from statistical frameworks to machine learning and deep learning approaches. These tools are essential for overcoming challenges related to data heterogeneity, scale, and interpretation. Ultimately, we aim to provide an overview of the current landscape of multi-omics integration in plant-pathogen research and to highlight future directions, including the expanding role of artificial intelligence in making sense of complex biological systems.

## Omics approaches in plant-pathogen interactions

**Fig. 2:**
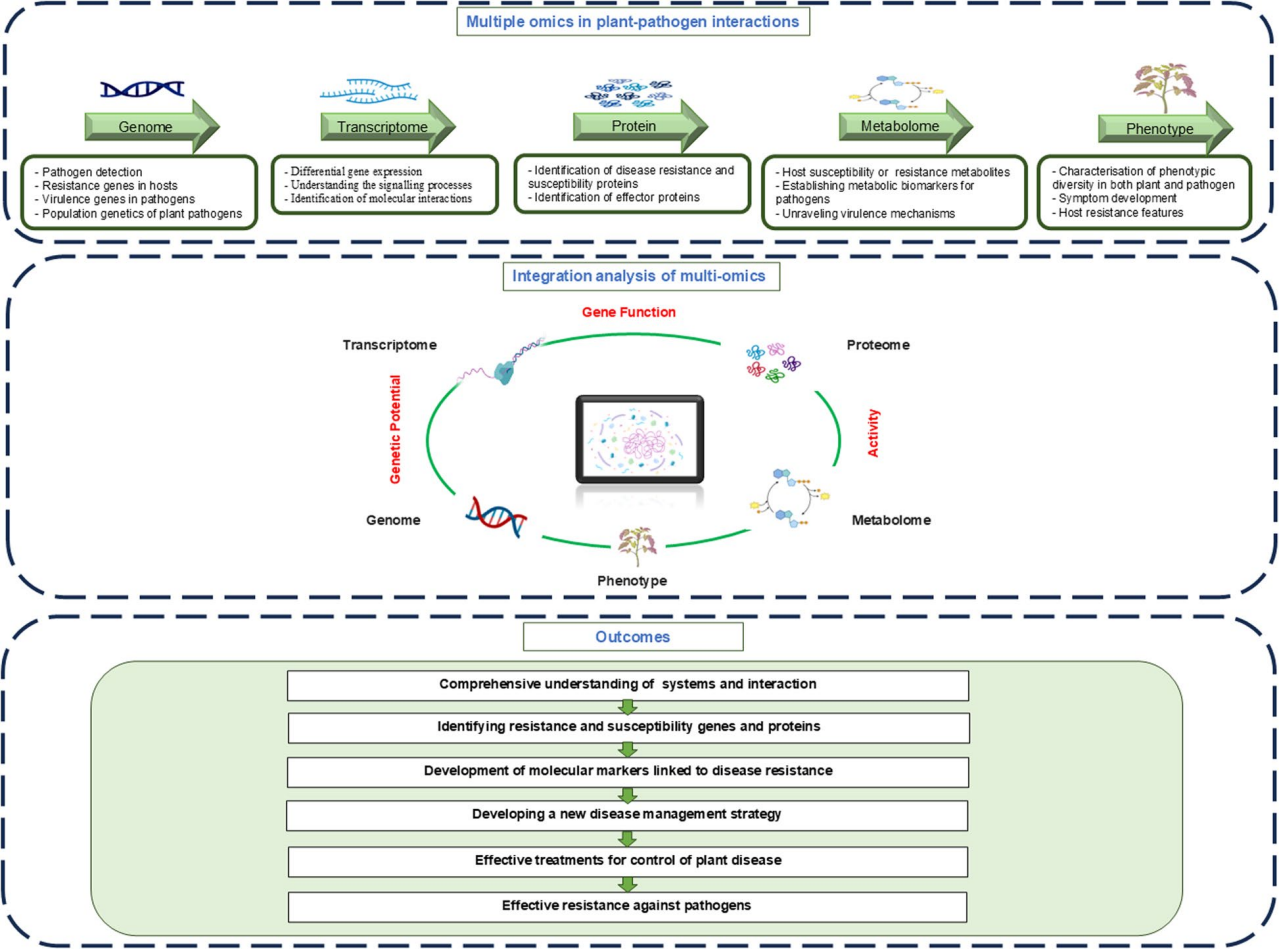
Multi-Omics in plant-pathogen interactions. This figure illustrates the integration of multi-omics approaches, including genomics, transcriptomics, proteomics, and metabolomics, in understanding plant-pathogen interactions. It highlights how these layers of analysis contribute to a comprehensive understanding of the mechanisms underlying plant responses to pathogens. The outcomes of multi-omics analyses are presented, showcasing their potential to identify key factors in disease resistance and susceptibility, inform breeding strategies, and enhance our understanding of the complex interactions between a plant and a pathogen

Pathogens employ diverse strategies to overcome plant defence mechanisms, plants, in turn, use various pathways to regulate their response to biotic stressors. Different omics tools are highly appropriate in elucidating these effects but most research into plant-pathogen interactions still focuses on a single type of omics data (Doni et al. 2022). Such dependence on a single-omic dataset runs the danger of offering only a partial picture of the dynamic interplay involved in plant-pathogen interactions. This may underlie the many reports of where pathogen virulence or plant resistance/susceptibility genes have only been partially validated in follow on studies (Naidoo et al. 2018). For instance, when Balotf et al. (2024) compared the defence response of potato roots to the soilborne pathogen *Spongospora subterranea*, it was observed that genes highly upregulated in resistant cultivars did not show corresponding increases in protein levels. In another experiment, Urquhart and Idnurm (2019) used CRISPR-Cas9 to disrupt 11 genes from *Leptosphaeria maculans* that were highly upregulated at the mRNA level during infection of canola (*Brassica napus*) plants but found that none of these genes were essential for fungal pathogenicity. Such misinterpretations can be avoided if a multi-omics approach is followed as it provides more complete pictures from integrated data from various biological layers (Ritchie et al. 2015). Figure 2 summarises the simplified concept of the layers of different levels of omics information, focusing on the regulatory patterns and principles governing how genetic materials influence phenotype. The following sections consider the pros and cons of single level omics approaches.

### Genomics

Genomics is the study of the structural and functional features of the genome of a given organism. The first generation of DNA sequencing techniques including the traditional Sanger sequencing method are shotgun-based techniques, while the next-generation sequencing methods are high-throughput techniques that are capable of sequencing thousands of reads simultaneously (Satam et al. 2023). Methods using e.g. the Illumina platform, tend to generate short reads, whilst third generation techniques, including Nanopore and PacBio, generate longer reads to ease the assembly of large genomes (Naidoo et al. 2018; Shendure et al. 2017). Whole genome sequencing of plants and their pathogens has invaluable in deciphering several aspects of plant-pathogen interactions including molecular mechanisms of host specificity in pathogenic interactions, virulence mechanisms, host resistance, and host susceptibility (Aylward et al. 2017; Dalio et al. 2017; Malapi-Wight et al. 2016).

The first plant genome sequenced, of Arabidopsis, was published in 2000 (Arabidopsis Genome Initiative 2000). Arabidopsis has a genome size of approximately 135 Mb, and according to the latest Araport11 re-annotation, it contains 27,655 protein-coding loci and 48,359 transcripts (Cheng et al. 2017). As of 2024, a total of 4,604 plant genomes from 1,482 plant species have been published (Bernal-Gallardo and de Folter 2024). The first complete genome for a plant pathogen was for the bacterial species *Xylella fastidiosa* (with a ~ 2,679 kb chromosome and two plasmids of ~ 51 and ~ 1.3 kb) (Simpson et al. 2000). Now, well-annotated genomes for plant-pathogenic bacteria, fungi, oomycetes and other pathogenic organisms have become available, and genomic data has been invaluable for the identification of resistance and virulence factors in plants and pathogens. Considering some of many possible examples, Pariyar et al. (2016) used genomic data to effectively identify the sources of resistance to a cyst nematode (*Heterodera filipjevi*) in wheat (*Triticum aestivum*) and Mhora et al. (2016) used genomic-based approaches to accurately predict disease resistance in lima beans *(Phaseolus lunatus)* against a downy mildew (*Phytophthora phaseoli*). Crucially, the derivation of high-quality genomic data underpins the assembly and annotation of sequence data for both transcriptomic and proteomics and therefore for multi-omics assessments.

### Transcriptomics

The transcriptome refers to the entirety of RNA molecules, including mRNA, small RNAs, and other RNA species within a tissue at a particular moment in time (Giacomello 2021). Methodologies for transcriptomics include RNA sequencing, RNA microarray, expressed sequence tag, and serial/cap analysis of gene expression (Lowe et al. 2017), of which RNA sequencing is the most advanced and has many advantages over other approaches (Wang et al. 2009). Gene expression analysis in plant pathology was first applied in 1999 to study potato-*Phytophthora infestans* interaction (Birch et al. 1999; Kamoun et al. 1999) and now many pathosystems have been assessed transcriptionally (Asai et al. 2014; Chen et al. 2023; De Cremer et al. 2013; Hayden et al. 2018; Meng et al. 2021; Molitor et al. 2011; Qiu et al. 2023; Schenk et al. 2012; Tan et al. 2009). Such studies have led to new insights into genes and pathways related to pathogen virulence or host resistance/susceptibility (Brown 2023).

By employing RNA sequencing, researchers can generate detailed profiles of gene expression at the specific tissue, highlighting the activation of pathogen-recognition receptors, and the signalling pathways involved in defence responses (such as those mediated by salicylic acid, jasmonic acid, and ethylene phytohormone pathways) (Luo et al. 2019). Transcriptomic analysis also provided deep insight into the modulation of genes involved in cell wall reinforcement, reactive oxygen species (ROS) production, and programmed cell death (Akbar et al. 2020). Additionally, transcriptomic studies helped uncover the role of effector molecules secreted by pathogens, which can manipulate host gene expression to suppress defence responses or promote virulence (Petitot et al. 2016). If validated at the functional level, these genes can be specifically targeted in breeding programs aiming to improve disease resistance (Yadav et al. 2024). Transcriptomic analysis of interacting plant and pathogen cells can lead to a more complete understanding of the signalling processes and molecular events influencing their association (Zhu et al. 2023).

Recent advancement in single-cell sequencing such as single-cell RNA sequencing enables the examination of gene expression at the individual cell level, rather than in whole tissue samples, which provides a clearer picture of the diversity within cell populations. For example, single-cell RNA sequencing can be applied to investigate how specific plant cells respond to pathogen infection, revealing gene expression patterns that are unique to particular cell types. Spatial RNA-seq also allows for the analysis of gene expression in individual cells while maintaining their precise location within the plant tissue (Yin et al. 2023). This approach offers valuable insights into the spatial arrangement of gene expression, both in the plant and the pathogen, during the infection process (Yin et al. 2023).

### Proteomics

Proteomics is the identification, quantification and characterisation of the protein content of a particular tissue, typically on a large scale and using unbiased or non-targeted techniques. The first studies to use proteomics, published in 1975, deployed 2-dimensional gel electrophoresis (2-DE) to study the proteome of *Escherichia coli* (O'Farrell 1975). In plants, proteomics can elucidate the functions of various proteins in developmental processes and growth, as well as reveal alterations in the proteome induced by biotic stresses such as plant pathogens. Proteomics has been used to study several important plant pathosystems and identify key proteins in plant-pathogen interactions (Gonzalez-Fernandez and Jorrin-Novo 2012; Liu et al. 2019), including those involved in host resistance or susceptibility, facilitating the development of more effective strategies for disease control management (Mehta et al. 2008).

There are numerous reports of using 2-DE to study host–pathogen interactions in plant pathology. For example, Kachroo et al. (1997), investigated the changes in the proteomes of *Magnaporthe oryzae* in the presence of extracts from resistant and susceptible rice cultivars. Other studies have used the 2-DE technique to analyse the proteomes of plant pathogens, identifying proteins related to both plant defence and pathogen virulence (Houterman et al. 2007; Kim et al. 2004; Rampitsch et al. 2006; Rep et al. 2004). Since these early reports, gel-based approaches have been largely superseded by gel-free or shotgun proteomics which utilises liquid chromatographic separation of high-complexity protein digests (Aebersold and Mann 2016).

Over the past two decades, mass spectrometry (MS), coupled with liquid chromatography (LC), has emerged as a leading technological platform for high-throughput identification and quantification of biomolecules in the “omics” sciences. Apart from genomics and transcriptomics, mass spectrometry is central to the high-throughput profiling of biomolecules. This is due to its exceptional sensitivity, the ability to separate analytes with high resolution by mass, rapid scanning speed, and the capability to obtain structural information through fragmentation (Holcapek et al. 2012). These features make mass spectrometry particularly well-suited for analysing complex mixtures with vast dynamic ranges (Shao and Lam 2017). In proteomic analysis, peptides are identified by peptide-spectrum matching (PSM), which requires a protein sequence database for the organism(s) under investigation. A search engine is used to computationally match the acquired data, specifically the MS2 spectra (fragments of the parental [full] molecule [MS1]), against the observable fragment ions from each potential matching peptide, which can be accurately predicted from the amino acid sequence (Rozanova et al. 2021). Proteomics search engines are increasingly augmented by deep-learning algorithms that consider retention time and ion mobility in addition to parameters such as peptide mass, charge state, and fragmentation pattern (Ng et al. 2023). In line with improvements in LC–MS technology and data acquisition techniques, shotgun proteomics has also become the most common method used for the large-scale study of proteins in plant pathology research (Balotf et al. 2022b; Tan et al. 2009). By enabling the simultaneous examination of thousands of proteins, shotgun proteomics provides valuable insights into the interactions between plants and pathogens, helping to uncover mechanisms of disease resistance and pathogen virulence (Elmore et al. 2021).

### Metabolomics

Small metabolite analysis can achieve insights into the metabolic profiling of many areas of plant studies such as growth, development, stress responses, and the discovery of phenotypic diversity induced by genetic variations or environmental disturbance (Schauer and Fernie 2006). Compared to other omics methodologies, metabolomics is considered a more precise assessment of molecular phenotype, as metabolite levels are often the summation of the biological processes occurring within organisms (Dettmer and Hammock 2004; Ryan and Robards 2006). Metabolomics studies are typically classified as either targeted or non-targeted (Han et al. 2023) and the most commonly used techniques are nuclear magnetic resonance (NMR) and the combination of either liquid or gas chromatography with MS. In some studies, these approaches are combined, providing complementary datasets and increasing the range of metabolite profiles.

Although the role of individual metabolites in plant diseases has been studied in different pathosystems for several decades (Morris et al. 1998), omics-scale metabolite profiling of plant-pathogen interactions was first reported by Allwood et al. (2006). They showed that phosphatidic acid and phosphatidyl glycerol phospholipids are major non-polar metabolites that distinguish *Brachypodium distachyon* response to *Magnaporthe grisea* infection. Later, Doehlemann et al. (2008) studied transcriptomic and metabolomic changes in maize induced by the biotrophic fungus *Ustilago maydis*. The increasing application of metabolomics in plant-pathogen research not only enhances our understanding of the biochemical responses to pathogens but also facilitates the identification of potential biomarkers for disease resistance (Allwood et al. 2021). This knowledge has significant implications for developing innovative strategies for crop protection and improving agricultural resilience.

Table 1 presents a broader range of selected research studies that utilised various omics approaches to better understand these interactions. These studies highlight the power of omics technologies in identifying key genes, proteins, and metabolites involved in plant defence mechanisms, as well as in revealing how pathogens manipulate host systems to facilitate infection.

**Table 1:** Selected examples of research work that used omics tools in the study of plant-pathogen interactions

Pathogen	Host	Omics type	Reference
**Single omics**			
*Fusarium graminearum*	Wheat	Genomics	(Goswami et al. 2006)
*Ralstonia solanacearum*	Potato	Genomics	(Ailloud et al. 2015)
*Heterodera filipjevi*	Wheat	Genomics	(Pariyar et al. 2016)
*Scleromitrula shiraiana*	Mulberry	Genomics	(Lv et al. 2020)
*Xanthomonas translucens*	Small grain cereals	Genomics	(Shah et al. 2021)
*Phytophthora infestans*	Potato	Transcriptomics	(Birch et al. 1999)
*Phytophthora infestans*	Potato	Transcriptomics	(Gyetvai et al. 2012)
*Sclerotinia sclerotiorum*	Pea	Transcriptomics	(Yajima and Kav 2006)
*Hyaloperonospora arabidopsidis*	Arabidopsis	Transcriptomics	(Asai et al. 2014)
*Rhizoctonia solani*	Wheat	Transcriptomics	(Hayden et al. 2018)
*Ralstonia solanacearum*	Tomato	Transcriptomics	(French et al. 2018)
*Magnaporthe oryzea*	Rice	Proteomics	(Kachroo et al. 1997)
*Clavibacter michiganensis*	Tomato	Proteomics	(Coaker et al. 2004)
*Verticillium dahlia*	Cotton	Proteomics	(Wang et al. 2011)
*Botryosphaeria dothidea*	Poplar	Proteomics	(Li et al. 2019a, b)
*Stagonospora nodorum*	Wheat	Metabolomics	(Tan et al. 2009)
*Botrytis cinerea*	Grapevine	Metabolomics	(Hong et al. 2012)
*Pseudomonas syringae*	Tobacco	Metabolomics	(Lee et al. 2013)
*Fusarium tucumaniae*	Soybean	Metabolomics	(Scandiani et al. 2015)
*Rhizoctonia solani*	Rice	Metabolomics	(Hu et al. 2017)
*Plasmopara viticola*	Grapevine	Metabolomics	(Negrel et al. 2018)
*Pyricularia oryzae*	Rice	Metabolomics	(Azizi et al. 2019)
*Colletotrichum theobromicola*	Strawberry	Metabolomics	(Dai et al. 2019)
**Multiple omics**			
*Phytophthora infestans*	Potato	Transcriptomics + Proteomics	(Ali et al. 2014)
*Cladosporium fulvum*	Tomato	Transcriptomics + Proteomics	(Karimi Jashni et al. 2020)
*Spongospora subterranea*	Potato	Transcriptomics + Proteomics	(Balotf et al. 2021, 2022a)
*Magnaporthe oryzea*	Rice	Transcriptomics + Proteomics	(Jeon et al. 2022)
*Ustilago maydis*	Maize	Transcriptomics + Metabolomics	(Doehlemann et al. 2008)
*Fusarium graminearum*	Wheat	Transcriptomics + Proteomics + Metabolomics	(Gunnaiah et al. 2012)

## Limitation of the single omics approach

The relationship between the genome and phenotype is complex, influenced by various factors that make it difficult to draw direct conclusions from genomic data alone. Transcriptomics, proteomics and metabolomics are key approaches used to investigate this relationship. The link between these levels is not straightforward. For example, not all mRNA transcripts are translated into proteins; some may be degraded, undergo alternative splicing, or be regulated at the translational level, reflecting the complexity of cellular responses to stress (McManus et al. 2015). Key elements influencing protein synthesis include protein half-life, delays in synthesis, and modulation of translation rates through regulatory interactions with non-coding RNAs like microRNAs (Liu et al. 2016). Despite significant advances, most transcriptomics studies focus primarily on protein-coding genes, often neglecting the roles of non-coding RNAs such as microRNAs and long non-coding RNAs (lncRNAs) (Ozsolak and Milos 2011), which play key regulatory roles. For example, miRNAs can bind to complementary sequences in mRNA molecules, leading to mRNA degradation or inhibition of translation, thus controlling which proteins are synthesised in response to stress signals (Makeyev and Maniatis 2008). Similarly, lncRNAs can interact with mRNAs, regulatory proteins, or components of the translation machinery to either enhance or inhibit protein synthesis, depending on the stress context (Gil and Ulitsky 2020). For instance, during heat shock, stress-induced lncRNAs can sequester or recruit RNA-binding proteins that regulate the stability and translation of mRNAs encoding HSPs and other protective factors (Xu et al. 2020; Statello et al. 2021). This regulation ensures a timely and coordinated response to stress, allowing cells to prioritise the translation of protective proteins while inhibiting the synthesis of non-essential proteins (Greenbaum et al. 2003; Koussounadis et al. 2015; Nakaminami et al. 2014). Research in human cell lines has suggested that mRNA abundance accounts for only 27% of the variability observed in protein abundance. In contrast, factors associated with translation and protein degradation can account for approximately 40% of the variability in protein levels (Vogel et al. 2010). Similarly, in maize, correlations between mRNA and protein levels across developing leaf segments were relatively low, ranging from 0.45 to 0.65, indicating that transcript levels alone explain only a portion of the variation in protein abundance, with post-transcriptional regulation likely playing a key role (Ponnala et al. 2014). However, it is important to note that such findings cannot be generalised across all datasets or conditions, as protein regulation is highly context dependent. Several factors influence these outcomes, such as the distribution of protein abundances, the mechanisms involved in post-translational modifications, and the conditions under which proteins are classified as"varied"(e.g., significantly altered in abundance or undergoing changes due to environmental stressors or signalling events) (Fu and Ares 2014). Additionally, factors such as translational efficiency, mRNA stability, protein half-life, and the role of molecular chaperones and degradation pathways (e.g., the ubiquitin–proteasome system and autophagy) play key roles in determining the final protein levels, which further complicates the picture (Das et al. 2021).

A technical limitation of transcriptomic data is the dependence on high-quality, well-annotated genomes. The accuracy and comprehensiveness of transcriptomic analyses heavily depend on the availability of a reference genome for the organism of interest, which serves as the basis for mapping and annotating the expressed genes (Bakkeren et al. 2016). The primary challenge for obligate biotrophic (non-culturable) plant pathogens is the difficulty of obtaining high molecular weight DNA for long-read DNA sequencing, which is essential for constructing high-quality, complete genomes. Unlike short-read sequencing methods, which may fail to capture the full complexity of a fungal genome due to the repetitive and often highly structured nature of fungal genomes, long-read sequencing provides the depth and continuity necessary for assembling complete genomes with fewer gaps and errors (Naranjo-Ortiz and Gabaldon 2020). Additionally, host contamination from the plant’s DNA can overshadow the pathogen sequences, making it difficult to selectively extract and sequence the fungal genome. It may be possible to obtain the genome for non-culturable pathogens from deep sequencing of RNA from infected plant tissue, but it can be difficult to obtain completed genomes by using these approaches.

Proteome data exhibit a more robust correlation with biological function compared to transcriptome data (Bludau and Aebersold 2020). However, a key challenge in proteomic analysis arises from the wide variation in protein concentrations within biological samples. These concentrations can differ significantly, causing low-abundance proteins to be masked by high-abundance proteins in complex mixtures (Righetti and Boschetti 2020). While this protein masking phenomenon is well reported in biofluids such as plasma (Anderson and Anderson 2002), in plant species, the highly abundant protein RuBisCO has a similar effect on limiting the depth of proteome coverage, specifically in leaf samples. Enriching low-abundance proteins by removing or depleting high-abundance proteins can help address this challenge (Jmeian and El Rassi 2009; Millioni et al. 2011) but can be technically challenging. Furthermore, the number of proteins identified from pathogens from infected plant samples is notably low. Numerous studies have aimed to detect pathogen-derived proteins within plant tissues, and whilst several thousand plant proteins have been identified only a few hundred are obtained from the pathogen (Balotf et al. 2021; Bindschedler et al. 2009; Zhou et al. 2006). This limitation poses significant challenges to understanding plant-pathogen interactions, as the low detection rate may hinder the identification of critical pathogen effectors and virulence factors.

In shotgun proteomics, the identification of peptides often results in a loss of the direct connection to the original proteins, making it difficult to distinguish between orthologous proteins from different species, such as a host and its pathogen in plant pathosystems. This challenge is particularly evident when two species share identical or highly similar peptide sequences (Hubler et al. 2019). For example, if two peptides that are assigned to a particular protein have identical sequences in the host and pathogen, it is not possible to unambiguously assign them to one species. However, if a third peptide unique to the host is identified, the principle of parsimony dictates that all three peptides are assigned to the host protein, disregarding the possibility that the pathogen variant may also be present (Gupta et al. 2015; Jayaraman et al. 2012). This is where transcriptomics provides an advantage, as mRNA sequencing can more easily differentiate between host- and pathogen-derived transcripts by detecting regions of the mRNA sequence that are species-specific.

Using metabolomics to study plant-pathogen interactions is also challenging, due to the complexity and diversity of metabolic compounds within such biological systems. Mass spectrometry is highly effective at detecting thousands of compounds in a single sample. However, no single technique can fully capture the entire metabolome because of the wide range of chemical properties in metabolites and the diversity of sample types (Ser et al. 2015). This limitation necessitates the development of numerous protocols, leading to inconsistencies in methodologies and complicating data reporting (Yanes et al. 2011). Additionally, the extraction of metabolites often leaves residual proteins in samples, which can skew results through post-extraction metabolite interconversions (House et al. 2024; Want et al. 2006). These challenges highlight the need for optimised extraction techniques and robust workflows to ensure an accurate representation of metabolic phenotypes in research.

Another challenge in sample analysis and data interpretation is the many shared metabolites between the pathogen and its host plant (Allwood et al. 2010; Camañes et al. 2015; Castro-Moretti et al. 2020; Pang et al. 2018). Metabolites often play central roles in multiple biochemical processes, making it challenging to accurately assign them to a single pathway. This promiscuity arises from the fact that many metabolites serve as intermediates or cofactors in different enzymatic reactions across diverse metabolic pathways, such as glycolysis, the tricarboxylic acid cycle, and amino acid metabolism. As a result, their roles may be context-dependent, influenced by cellular conditions or the presence of specific enzymes. Therefore, accurate determination of the origin of the extracted metabolites, whether it originates from the host plant or the pathogen, presents a significant challenge (Munoz-Hoyos and Stam 2023; Pang et al. 2018) and ideally would use of isotopically labelled metabolomes in one of the interacting partners (Allwood et al. 2021). Additionally, the availability of a comprehensive database is crucial for the success of metabolomics, as metabolomics software increasingly relies on matching data to spectral libraries based on ion-fragmentation data (usually, MS2). Although a valid approach, this metabolite library-based approach prioritises the identification of known over novel metabolites. The absence of specific, comprehensive databases and libraries for the host plants and their pathogens poses a significant challenge in metabolomics analysis (Castro-Moretti et al. 2020).

A lack of correlation between metabolomics and other omic levels especially transcriptomics has been reported in several studies. This can be explained by feedback inhibition mechanisms which prevent excessive accumulation of discrete metabolites and preserve homeostasis (Naz et al. 2023; Sander et al. 2019). In instances of feedback inhibition, the final metabolite may be reduced despite the high expression of genes at the transcriptome level (Goyal et al. 2010). Therefore, multi-omic strategies are required as transcriptome and metabolite levels cannot be mutually inferred.

In summary, single-omics techniques, while powerful, each have notable limitations. Transcriptomics may not accurately reflect protein abundance due to regulatory processes like alternative splicing and microRNA-mediated control. Proteomics can be biased by highly abundant proteins, making it difficult to detect low-abundance proteins or distinguish between host and pathogen proteins. Metabolomics also faces challenges in capturing the full range of metabolites, with shared metabolites between host and pathogen complicating the analysis. These issues highlight the need for multi-omics approaches to provide a more complete and integrated view of biological systems.

## Using interactive omics approaches

By integrating data from various omics layers researchers can gain deeper insights into biological processes, and the molecular interactions that drive phenotypic outcomes. In plant-pathogen interactions, multi-omics can enable the identification and annotation of effector proteins which are crucial for understanding how pathogens manipulate host plant defence and can reveal key factors that contribute to disease resistance or susceptibility (Kahar et al. 2024). Multi-omics can also help marker identification for early disease diagnosis. The corollary to this is the availability of methods that can overcome the challenge of integrating different levels of omics data. The following discussion provides an overview of some key tools that facilitate the integration and analysis of diverse omics datasets.

One notable example of this is the R package mixOmics (Rohart et al. 2017) which employs various statistical techniques to analyse and correlate data from different omics layers, making it particularly useful for studying complex biological systems like plant-pathogen interactions. The package provides tools for multivariate analysis, including canonical correlation analysis (CCA) (González et al. 2008), sparse partial least squares (sPLS) (Chun and Keles 2010), and sparse partial least squares discriminant analysis (sPLS-DA) (Boulesteix 2004). These methods enable researchers to explore the relationships between different omics datasets, identify key variables contributing to the interaction, and gain insights into the underlying biological processes.

Multi-omics factor analysis (MOFA) is another tool that is capable of extracting and correlating factors across multiple omics datasets. MOFA is designed to handle heterogeneity and missing data, common challenges in omics studies. By employing a probabilistic framework, MOFA models the latent factors that best explain the observed data while accounting for uncertainties (Argelaguet et al. 2018). By identifying shared factors across different omics layers, MOFA helps in understanding the underlying biological processes and the interaction between plants and their pathogens at a systems level. In addition to integration at the level of individual components, bioinformatic approaches can be used for network- and pathway-level integration of multiple omics datasets. Tools like Cytoscape (Smoot et al. 2011), Pathway Commons (Cerami et al. 2010) and ReactomeGSA (Griss et al. 2020) enable researchers to visualise and analyse molecular interaction networks. These tools can be used to integrate omics data by mapping proteins, genes, and metabolites onto known biological networks, thereby revealing how changes in one layer of data might influence other layers.

Causal weighted gene co-expression network analysis (CWGCNA) is an extension of the traditional weighted gene co-expression network analysis (WGCNA) that integrates causal inference models to explore the directional relationships between gene co-expression modules and phenotypic traits. While WGCNA identifies gene modules based on their co-expression patterns, CWGCNA goes further by incorporating causal mediation analysis to determine whether changes in gene expression drive phenotype alterations or whether phenotypic changes influence gene expression. This causal framework provides a more dynamic understanding of gene-phenotype interactions and enables researchers to uncover the molecular mechanisms behind complex traits and diseases. CWGCNA is particularly valuable in studies of diseases, where it helps identify potential biomarkers by illuminating the cause-and-effect relationships between genes and phenotypes (Liu 2024).

The integration of multi-omics data is a powerful yet computationally intensive endeavour, particularly in the context of plant-pathogen interactions where high-dimensional, heterogeneous datasets are generated. Each omics layer differs in scale, data structure, and noise profile, necessitating robust computational methods to harmonise and interpret them. A major challenge lies in the pre-processing and normalisation of data from diverse platforms (Jan et al. 2025). This involves removing batch effects, handling missing values, and scaling data appropriately across omics layers, all of which are resource-demanding when working with large sample sizes or time-course experiments (Goh et al. 2017; Schumann et al. 2025). In addition, data integration methods such as similarity network fusion, Bayesian inference, or latent factor models (e.g., MOFA2, iClusterPlus) require substantial computing power and memory, particularly as the number of samples and features increases (Subramanian et al. 2020).

High-performance computing (HPC) environments or cloud-based platforms are often essential for executing these analyses. For instance, multi-omics integration using DIABLO (Singh et al. 2019) from the mixOmics R package or training deep learning models for feature extraction may take several hours to days, depending on the complexity of the dataset and the parameters used. In smaller laboratories, these requirements may exceed available infrastructure, highlighting the need for collaborative efforts or cloud-based alternatives such as Galaxy (Afgan et al. 2018), Terra (Li et al. 2020), or CyVerse (https://cyverse.org/).

Moreover, many integration tools require advanced scripting in R or Python and rely on complex dependencies, posing additional barriers for researchers without computational training (Morabito et al. 2025). Therefore, the success of multi-omics studies is closely linked not only to experimental design but also to access to adequate computational infrastructure and interdisciplinary expertise.

## Role of artificial intelligence in multi-omics data integration

If the rapid expansion of multi-omics data presents both an opportunity and a challenge (Murmu et al. 2024), artificial intelligence (AI), particularly machine learning (ML) and deep learning (DL) techniques, has emerged as a powerful solution to this challenge (Kang et al. 2022; Yan and Wang 2023). These approaches can uncover hidden patterns, prioritise key molecular features, and support predictive modelling across diverse omics layers (Reel et al. 2021).

In the context of plant-pathogen systems, AI tools have been applied to tasks such as disease prediction, gene function annotation, biomarker discovery, and resistance gene identification (Bai et al. 2024). For instance, ML techniques such as random forests (Acharjee et al. 2016) and support vector machines (Kim et al. 2017) can be used to build predictive models that incorporate information from multiple omics layers. These models can assist in predicting plant responses to pathogen attacks, identifying novel resistance genes, and uncovering new targets for crop improvement (Kimotho and Maina 2024). Algorithms like support vector machines (SVMs), and k-nearest neighbours (k-NN) have been employed to classify resistant and susceptible phenotypes based on integrated transcriptomic and metabolomic profiles (Lin and Lane 2017; Valous et al. 2024). Deep learning models, such as deep neural network genomic prediction (DNNGP), have shown potential in reducing dimensionality and capturing non-linear relationships within multi-omics datasets in plants (Wang et al. 2023).

Despite these advances, AI integration in plant multi-omics remains in its infancy. Challenges such as data heterogeneity, poor recording of crucial metadata, small sample sizes, and the need for domain-specific model training persist. Nonetheless, the trajectory is promising, especially as public multi-omics databases grow and interdisciplinary collaborations between plant biologists and data scientists become more common.

## Conclusion and future directions

It is our assertion that the interactions between plants and pathogens are profoundly complex and cannot be fully understood through a single omics approach alone. Integrating multiple omics layers into a multi-omics approach addresses these limitations by providing a more comprehensive view of the biological system. The integration of omics data is not without its challenges. Variability in data completeness and quality, differences in data scales, and the complexity of biological systems can complicate the analysis. Therefore, it is essential to use robust statistical and computational methods to address these issues (Canzler et al. 2020; Gomez-Cabrero et al. 2014). At the raw data level, integrating data from different omics platforms involves correlating patterns across diverse data types (Gomez-Cabrero et al. 2014). This process is complicated by variations in experimental metadata and data formats, measurement scales, and noise levels. Advanced computational methods, such as dimensionality reduction and machine learning, need to address these challenges. However, training models and algorithms to recognise patterns across heterogeneous datasets requires significant computational resources and expertise (Picard et al. 2021). Therefore, addressing the challenges of data integration at both the raw data and pathway levels will continue to be essential for advancing research and practical applications in plant disease management.

AI-based integrative analyses could facilitate the integration of multiple omics data by aligning heterogeneous omics data through advanced feature extraction and fusion strategies. For example, deep generative models can be used to infer missing omics layers or to simulate pathogen responses under different environmental conditions (Rivero-Garcia et al. 2024; Yan and Wang 2023). In combination with network-based approaches, AI helps construct dynamic regulatory and interaction networks that reflect real-time changes during infection processes (Yan et al. 2018; Cembrowska-Lech et al. 2023). Despite the promising potential of AI in multi-omics integration, there are key areas that require further development. To fully harness the power of AI, future studies must focus on transparent model interpretation, ensuring that the underlying processes are understandable and replicable. Robust benchmarking standards should be established to evaluate AI methods, allowing researchers to assess the performance and reliability of AI-driven analyses. Furthermore, the development of plant-specific AI tools is essential, as current models and algorithms often lack tailored solutions that account for the unique characteristics of plant biology and pathogen interactions. By addressing these gaps, AI can become a more effective tool in advancing plant science, especially in understanding complex, multi-dimensional interactions such as those seen in plant-pathogen interactions.

**Fig. 3:**
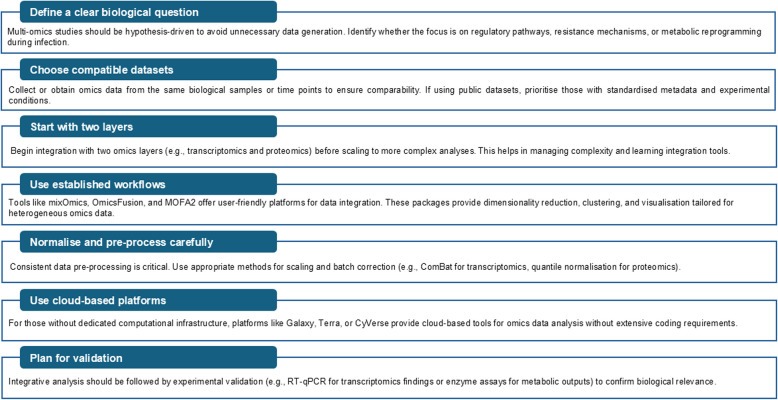
A practical framework for initiating multi-omics integration in plant-pathogen interaction studies

For researchers aiming to incorporate multi-omics approaches into their plant-pathogen studies, Fig. 3 presents a structured workflow outlining key steps for dataset selection, integration strategies, and best-practice methodologies to facilitate effective implementation. In summary, while significant progress has been made, continued innovation and collaboration across disciplines are essential to overcome the challenges of multi-omics integration. By leveraging these advancements, our understanding of plant-pathogen dynamics can be enhanced to better contribute to the development of more effective solutions for crop protection.

## Data Availability

Not applicable.

## Abbreviations

AI: Artificial Intelligence
CCA: Canonical Correlation Analysis
DL: Deep Learning
DNNGP: Deep Neural Network Genomic Prediction
HSP: Heat Shock Proteins
k-NN: K-Nearest Neighbours
LC: Liquid Chromatography
MAPK: Mitogen-Activated Protein Kinase
MOFA: Multi-Omics Factor Analysis
MS: Mass Spectrometry
ML: Machine Learning
NMR: Nuclear Magnetic Resonance
PAMP: Pathogen-Associated Molecular Patterns
PSM: Peptide-Spectrum Matching
ROS: Reactive Oxygen Species
RuBisCO: The enzyme ribulose-1,5-bisphosphate carboxylase/oxygenase
sPLS: Sparse Partial Least Squares
SVMs: Support Vector Machines
WGCNA: Weighted Gene Co-expression Network Analysis
